# Genome of ‘Charleston Gray’, the principal American watermelon cultivar, and genetic characterization of 1,365 accessions in the U.S. National Plant Germplasm System watermelon collection

**DOI:** 10.1111/pbi.13136

**Published:** 2019-05-07

**Authors:** Shan Wu, Xin Wang, Umesh Reddy, Honghe Sun, Kan Bao, Lei Gao, Linyong Mao, Takshay Patel, Carlos Ortiz, Venkata L. Abburi, Padma Nimmakayala, Sandra Branham, Pat Wechter, Laura Massey, Kai‐Shu Ling, Chandrasekar Kousik, Sue A. Hammar, Yaakov Tadmor, Vitaly Portnoy, Amit Gur, Nurit Katzir, Nihat Guner, Angela Davis, Alvaro G. Hernandez, Chris L. Wright, Cecilia McGregor, Robert Jarret, Xingping Zhang, Yong Xu, Todd C. Wehner, Rebecca Grumet, Amnon Levi, Zhangjun Fei

**Affiliations:** ^1^ Boyce Thompson Institute Cornell University Ithaca NY USA; ^2^ Department of Biology West Virginia State University Institute WV USA; ^3^ National Engineering Research Center for Vegetables Beijing Academy of Agriculture and Forestry Sciences Key Laboratory of Biology and Genetic Improvement of Horticultural Crops (North China) Beijing China; ^4^ Department of Biochemistry and Molecular Biology Howard University College of Medicine Washington DC USA; ^5^ Horticultural Science Department North Carolina State University Raleigh NC USA; ^6^ U.S. Department of Agriculture‐Agricultural Research Service U.S. Vegetable Laboratory Charleston SC USA; ^7^ Department of Horticulture Michigan State University East Lansing MI USA; ^8^ Department of Vegetable Research Agricultural Research Organization Newe Ya'ar Research Center Ramat Yishay Israel; ^9^ Sakata Seed America Fort Myers FL USA; ^10^ Sakata Seed America Woodland Research Station Woodland CA USA; ^11^ Roy J. Carver Biotechnology Center University of Illinois at Urbana‐Champaign Urbana IL USA; ^12^ Department of Horticulture University of Georgia Athens GA USA; ^13^ U.S. Department of Agriculture‐Agricultural Research Service Plant Genetic Resources Conservation Unit Griffin GA USA; ^14^ Syngenta Beijing Innovation Center Beijing China; ^15^ U.S. Department of Agriculture‐Agricultural Research Service Robert W. Holley Center for Agriculture and Health Ithaca NY USA

**Keywords:** watermelon, ‘Charleston Gray’, genome sequence, genetic diversity, *Citrullus* germplasm, genotyping‐by‐sequencing, genome‐wide association study, disease resistance

## Abstract

Years of selection for desirable fruit quality traits in dessert watermelon (*Citrullus lanatus*) has resulted in a narrow genetic base in modern cultivars. Development of novel genomic and genetic resources offers great potential to expand genetic diversity and improve important traits in watermelon. Here, we report a high‐quality genome sequence of watermelon cultivar ‘Charleston Gray’, a principal American dessert watermelon, to complement the existing reference genome from ‘97103’, an East Asian cultivar. Comparative analyses between genomes of ‘Charleston Gray’ and ‘97103’ revealed genomic variants that may underlie phenotypic differences between the two cultivars. We then genotyped 1365 watermelon plant introduction (PI) lines maintained at the U.S. National Plant Germplasm System using genotyping‐by‐sequencing (GBS). These PI lines were collected throughout the world and belong to three *Citrullus* species, *C. lanatus*,* C. mucosospermus* and *C. amarus*. Approximately 25 000 high‐quality single nucleotide polymorphisms (SNPs) were derived from the GBS data using the ‘Charleston Gray’ genome as the reference. Population genomic analyses using these SNPs discovered a close relationship between *C. lanatus* and *C*. *mucosospermus* and identified four major groups in these two species correlated to their geographic locations. *Citrullus amarus* was found to have a distinct genetic makeup compared to *C. lanatus* and *C*. *mucosospermus*. The SNPs also enabled identification of genomic regions associated with important fruit quality and disease resistance traits through genome‐wide association studies. The high‐quality ‘Charleston Gray’ genome and the genotyping data of this large collection of watermelon accessions provide valuable resources for facilitating watermelon research, breeding and improvement.

## Introduction

Watermelon (*Citrullus lanatus*) is an important crop consumed throughout the world and is a rich source of lycopene, citrulline and other human health‐promoting compounds (Perkins‐Veazie *et al*., 2007). Nearly six per cent of all land used for growing vegetables is planted with watermelon, which had an annual global production of 117 million tonnes in 2016 (FAOSTAT, http://www.fao.org/faostat/en/). China is the largest producer and consumer of watermelon with 70 million tonnes produced per year. China and the United States both have intensive watermelon breeding programmes to meet varied consumer preferences. For example, cultivars of East Asian ancestry mainly produce small to mid‐sized globular fruits with a thin rind, while American cultivars tend to produce large, oblong fruits with a thick rind. Differential selection pressures in the two regions have led to distinct East Asian and American watermelon ecotypes (Guo *et al*., 2013; Sheng *et al*., 2012). Currently, the only published watermelon reference genome was constructed using the Chinese elite line ‘97103’, representing the East Asian type (Guo *et al*., 2013). Recent comparative genomic and pan‐genome studies have highlighted the importance of structural variations (SVs), particularly presence–absence variations (PAVs) and copy number variations (CNVs) (Gao *et al*., 2019; Zhang *et al*., 2015). Quantitative trait locus (QTL) and association mapping studies with American germplasm using the ‘97103’ genome as the reference would be limited to the genetic makeup of a single East Asian cultivar, potentially missing causal variants.

A large number of watermelon cultivars have been developed in the United States since the mid‐19th century (Levi *et al*., 2001), including ‘Charleston Gray’ which is considered the principal American dessert watermelon cultivar. ‘Charleston Gray’ was released in 1954 and was the most popular commercially grown watermelon in the United States for more than a decade. It produces large oblong fruits with a light green exterior, pink‐red flesh, and a thick and tough rind, as required for long‐distance shipping. It also harbours resistance to the soil‐borne disease Fusarium wilt and the foliar disease anthracnose. ‘Charleston Gray’ has been used in numerous watermelon breeding programmes (Wehner, 1999) and in the development of many improved cultivars. Therefore, unlocking the genome of ‘Charleston Gray’ would further facilitate watermelon breeding.

Many years of cultivation and selection for desirable fruit qualities have resulted in an overall narrowing of the genetic base among sweet dessert watermelon cultivars, and consequently contributed to their susceptibility to a large number of diseases and pests (Levi *et al*., 2001, 2017). As a result, there is a continual need to expand the genetic base of watermelon cultivars and enrich them with alleles conferring resistance to biotic stresses such as diseases, insects and nematodes in addition to alleles associated with desirable fruit quality. The *Citrullus* genus, whose centre of origin is Africa, includes seven recognized species (Chomicki and Renner, 2015). Two of these species, *C*. *amarus* (also known as citron) and *C*. *mucosospermus* (also known as egusi melon), were until recently considered to be subspecies of *C. lanatus* (Chomicki and Renner, 2015), and they can cross readily with cultivated watermelon. *Citrullus lanatus* is thought to be native to northern Africa, *C. mucosospermus* to sub‐Saharan western Africa, and *C. amarus* to southern Africa (Jarret *et al*., 1997; Levi *et al*., 2017; Paris, 2015). Unlike *C. lanatus*,* C. mucosospermus* is primarily cultivated for oil‐rich seeds. *Citrullus mucosospermus* and *C. amarus* are valuable sources of resistance to many diseases including Phytophthora fruit rot, powdery mildew, Fusarium wilt, gummy stem blight, anthracnose and various viruses (Levi *et al*., 2017). Therefore, these highly interfertile species have the potential to provide valuable alleles to broaden the genetic base of watermelon and increase disease and pest resistance in elite cultivars.

The U.S. National Plant Germplasm System (NPGS; https://www.ars-grin.gov/npgs/index.html) maintains a large collection of *Citrullus* accessions as Plant Introductions (PIs) collected/acquired from different geographic regions in Africa and throughout the world. The PIs within this *Citrullus* collection exhibit great phenotypic and genetic diversity (Levi *et al*., 2013; Nimmakayala *et al*., 2014) and thus represent a valuable resource for discovery of unique qualities useful for improving fruit nutritional contents and resistance to biotic and abiotic stresses in watermelon cultivars. Elucidating the genetic diversity and relationships among these PIs is an important step towards the identification of QTLs associated with beneficial attributes.

In this study, we sequenced and *de novo* assembled a high‐quality genome of the principal American watermelon cultivar ‘Charleston Gray’, as a complement to the genome of ‘97103’, an East Asian cultivar. Comparative genomic analyses of ‘Charleston Gray’ and ‘97103’ identified a number of genome variations overlapping with QTLs of important traits such as fruit weight and shape. We then genotyped 1365 *Citrullus* accessions from the NPGS (including *C. lanatus*,* C. amarus* and *C. mucosospermus*) using genotyping‐by‐sequencing (GBS; Elshire *et al*., 2011). Single nucleotide polymorphisms (SNPs) were called from the GBS data using the ‘Charleston Gray’ genome as the reference and were subsequently used to assess genetic diversity, phylogenetic relationships and population structure among these *Citrullus* accessions. The SNP set was also used to perform genome‐wide association studies (GWAS) for the identification of genomic regions associated with several important fruit quality and disease resistance traits.

## Results

### Genome sequencing, assembly, anchoring and quality evaluation

The ‘Charleston Gray’ genome was sequenced using the Illumina technology, which produced a total of 95.5 Gb of high‐quality cleaned sequences from paired‐end and mate‐pair libraries with insert sizes ranging from 400 bp to 20 kb (Table S1). These sequences represented approximately a 228× coverage of the ‘Charleston Gray’ genome with an estimated size of 419.2 Mb based on the k‐mer depth distribution analysis of the sequencing reads (Figure S1). *De novo* assembly yielded a draft genome of 396.4 Mb, representing 94.6% of the ‘Charleston Gray’ genome. The assembly consisted of 21 498 contigs and 2034 scaffolds (>500 bp), with N50 sizes of 36.7 kb and 7.47 Mb, respectively, and 90% of the genome draft was assembled into only 60 scaffolds (Table 1). The ‘Charleston Gray’ assembly had higher contiguity compared with that of the Chinese elite watermelon line ‘97103’ (Guo *et al*., 2013), as indicated by increased N50 and N90 scaffold lengths (Table 1).

**Table 1:** Assembly statistics of watermelon ‘Charleston Gray’ and ‘97103’ genomes

	Charleston Gray				97103			
	Scaffold*		Contig		Scaffold*		Contig	
	Size (bp)	Number	Size (bp)	Number	Size (bp)	Number	Size (bp)	Number
Longest	23 422 029	1	235 198	1	8 716 783	1	227 474	1
N50	7 471 260	17	36 674	3073	2 378 183	42	26 377	3316
N90	1 615 926	60	9605	10 630	374 692	184	3971	15 057
Total	396 351 412	2034	375 815 318	21 498	353 466 419	1793	321 373 230	43 342

* Scaffolds longer than 500 bp in size were included in the assembly.

**Figure 1:**
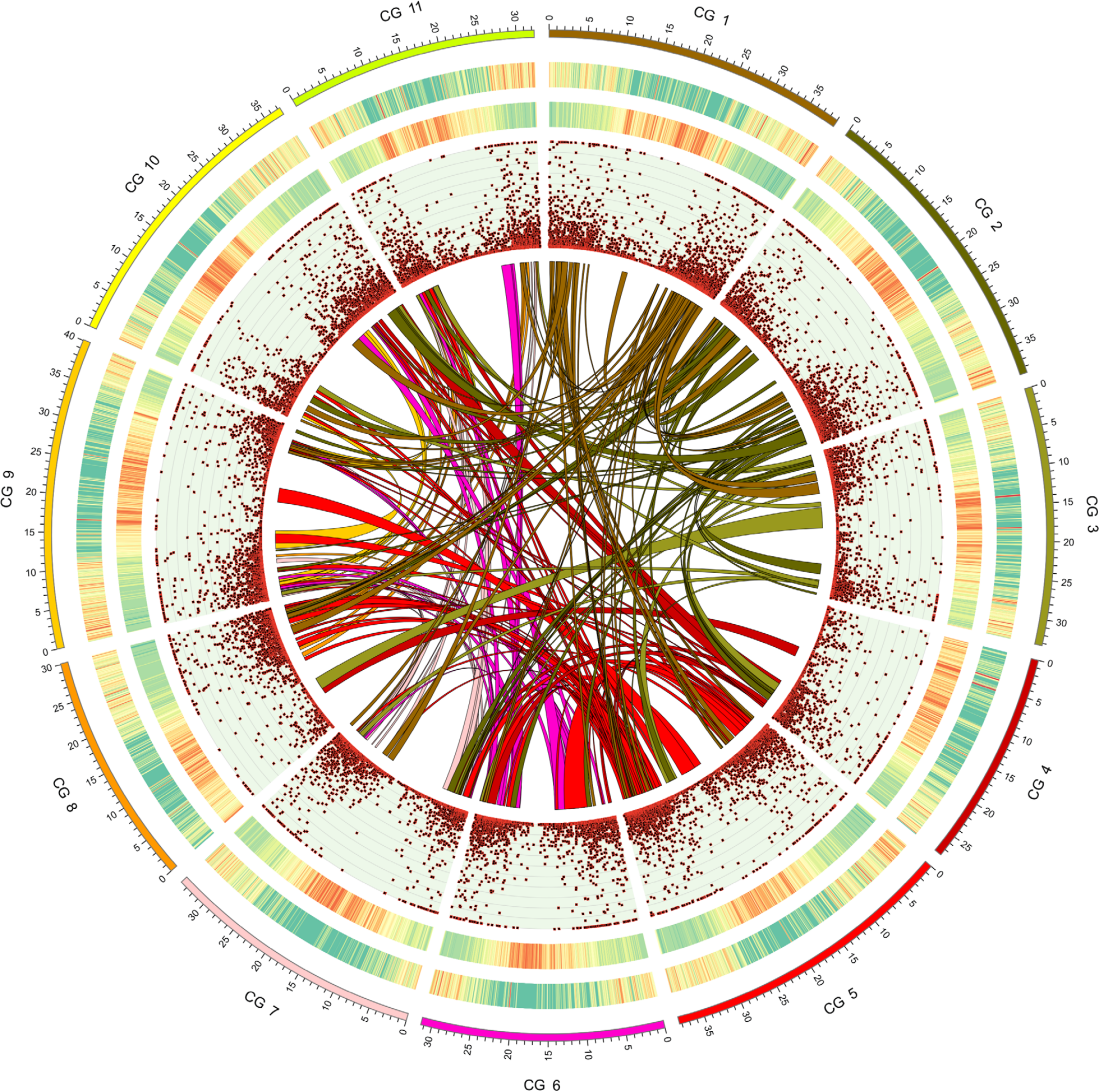
Genomic landscape of watermelon, ‘Charleston Gray’. The outermost circle is the ideogram of 11 chromosomes in Mb scale, followed by circles of gene density and TE density represented by percentage of genomic regions covered by genes and repeat sequences in 200‐kb windows, respectively (green to red, low to high), gene expression levels (RPKM; Maximum = 200) and syntenic blocks within the genome depicted by lines.

Using an integrated high‐density genetic map (Ren *et al*., 2014), 90.3% (378.7 Mb) of the ‘Charleston Gray’ assembly were anchored to 11 linkage groups, and 90.5% of the anchored scaffolds (342.8 Mb) were oriented. We assigned orientation to an additional of 9.9 Mb of scaffolds, according to two other recently developed genetic maps (Branham *et al*., 2017; Reddy *et al*., 2014). Furthermore, based on the genome synteny between ‘97103’ and ‘Charleston Gray’, an additional 26.6 Mb of the ‘Charleston Gray’ scaffolds were oriented, and 3.8 Mb of scaffolds were anchored. One misassembled scaffold was identified according to the genetic map and mate‐pair read alignments, and was broken into two scaffolds. Finally, we obtained a chromosome‐scale assembly of the ‘Charleston Gray’ genome, of which 382.5 Mb (96.2% of the total assembly) in 100 scaffolds were constructed into 11 pseudomolecules and 379.2 Mb (95.4% of the assembly) were oriented (Table S2 and Figure 1).

To evaluate the quality of the assembly, we first aligned RNA‐Seq reads and ESTs (expressed sequence tags; Levi *et al*., 2006) to the ‘Charleston Gray’ genome. Up to 94% of the RNA‐Seq reads were mapped to the genome, and more than 95% of the ESTs were covered by the genome (Table S3). The completeness of the ‘Charleston Gray’ assembly was further assessed with BUSCO (Simão *et al*., 2015), which showed that 91.8% of the core conserved plant genes were completely covered by the assembly, while another 1.7% were partially covered (Table S4). Together, these results confirmed the high quality of the ‘Charleston Gray’ assembly.

### Comparative genomic analysis of ‘Charleston Gray’ and ‘97103’

We identified 194.9 Mb of repeat sequences in the ‘Charleston Gray’ genome, contributing to 51.7% of the genome (Table S5), which was higher than the 45.2% repetitive content in the ‘97103’ genome (Guo *et al*., 2013), mainly attributed to a better assembly of repeat regions in the ‘Charleston Gray’ genome. A total of 22 546 protein‐coding genes were predicted in ‘Charleston Gray’, among which 18 982 genes (84.2%) were assigned with biological functions. Comparison between orthologous genes in ‘Charleston Gray’ and ‘97103’ revealed a chromosome‐level syntenic relationship (Figure S2). Most differences between the two assemblies were observed around the centromeric regions (Figure S3). For example, the ‘big inversion’ on chromosome 1 might not be an actual SV between the two genomes but was likely caused by incorrect anchoring in the ‘97103’ genomes as a result of uncertainty in ordering short scaffolds within a close genetic distance (Figure S4a). In this case, the assembly of the ‘Charleston Gray’ scaffolds 15 and 19 spanning the ‘inversion’ breakpoints was well supported by the mate‐pair reads (Figure S4b), which suggested that, with a higher contiguity, the ‘Charleston Gray’ assembly provided more reliable pseudomolecule structures of the watermelon genome for facilitating QTL mapping and molecular genetic studies.

A total of 214 113 SNPs and 16 052 small indels were identified between the ‘Charleston Gray’ and ‘97103’ genomes, of which 184 567 SNPs and 13 664 small indels were located in the intergenic regions, and 29 546 SNPs and 2388 small indels were located in the introns of genes (23 947 SNPs and 2275 indels) or coding regions (5599 SNPs and 113 indels) (Table S6). Among genes with SNPs in the coding regions, 1810 had nonsynonymous mutations, 80 lost start or stop codons, and 1506 had synonymous mutations. We further identified a total of 3766 high‐confidence large SVs (≥50 bp) between the ‘Charleston Gray’ and ‘97103’ genomes, including 2913 indels and 853 tandem/repeat contractions/expansions (Table S7). The majority of these SVs (88.8%) were less than 1 kb in size (Figure S5). About 81.5% (3069) of the SVs were located in intergenic regions, 518 in introns of genes, and 179 affected the coding sequences (Table S7). A total of 815 SVs were found to overlap with 52 QTLs known to affect watermelon fruit traits (Table S8), and 400 were located within fruit shape and fruit weight QTLs (Table S8), which might be associated with the more elongated fruit shape and larger fruit size of ‘Charleston Gray’ as compared to ‘97103’, such as the 159‐bp deletion in the *ClFS1* gene (ClCG03G016090) of ‘Charleston Gray’ known to be associated with elongated fruit shape (Dou *et al*., 2018).

### Genotyping of the watermelon germplasm collection and variation identification

**Figure 2:**
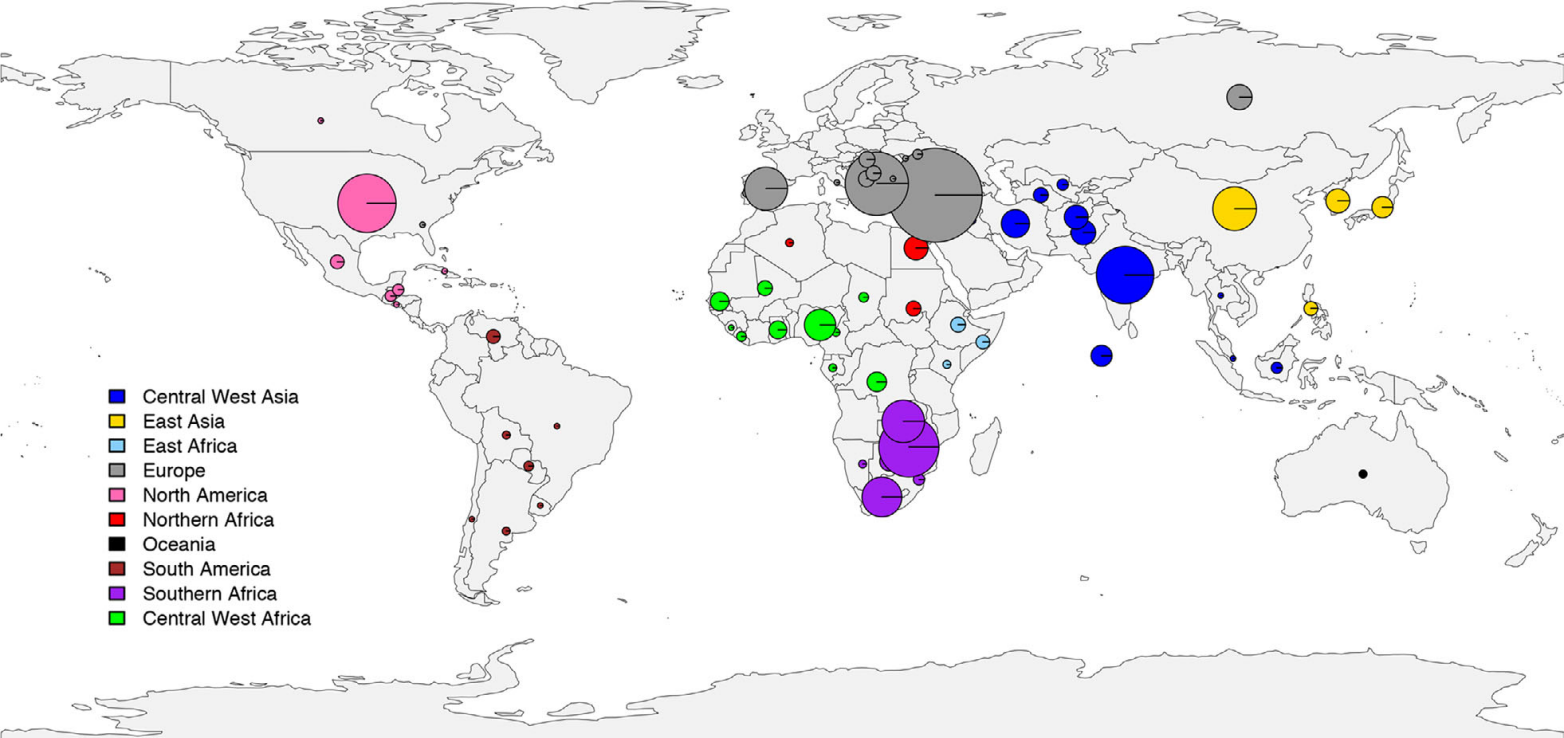
Geographical distribution of the 1365 *Citrullus* spp. accessions in the National Plant Germplasm System. The diameter of the circle is proportional to the number of accessions from each country.

To facilitate the utilization of the *Citrullus* spp. germplasm stored in the NPGS for watermelon improvement, we performed GBS on 1365 *Citrullus* PIs collected throughout the world (Figure 2), including 1211 *C. lanatus*, 52 *C*. *mucosospermus* and 102 *C. amarus* PIs (Table S9). Most *C. lanatus* PIs are modern cultivars and are the result of many years of domestication and selection for desirable fruit qualities that do not exist in their closely related citron or egusi type watermelons. The *C. lanatus* and *C*. *mucosospermus* PIs in this study were collected in East Asia, North and South America, Europe, Central and West Asia, Oceania and different regions in Africa, while the majority of *C. amarus* PIs were collected in their natural surroundings in southern Africa (Figure 2 and Table S9).

A total of ~1.0 billion reads of 101 bp in length were generated using GBS, from which 388 293 unique tags with at least 10 read counts were obtained, corresponding to ~0.8 billion reads, and used for SNP calling. About 49.5% and 16.5% of these reads were aligned to unique and multiple positions in the ‘Charleston Gray’ genome, respectively, and the unaligned reads were mainly from the chloroplast and mitochondrion genomes. Approximately 2.4% of the ‘Charleston Gray’ genome was covered by GBS reads, which is typical for GBS data (Elshire *et al*., 2011). A total of 61 520 SNPs were identified, among which 25 308 were biallelic with a minor allele frequency (MAF) >0.01 and a missing data rate ≤50%, and were used in the subsequent phylogenetic and population structure analyses. These SNPs were distributed across the ‘Charleston Gray’ genome with an average of one SNP per 15.7 Kb (Figure S6).

### Phylogenetic relationships and population structure of *Citrullus* accessions

**Figure 3:**
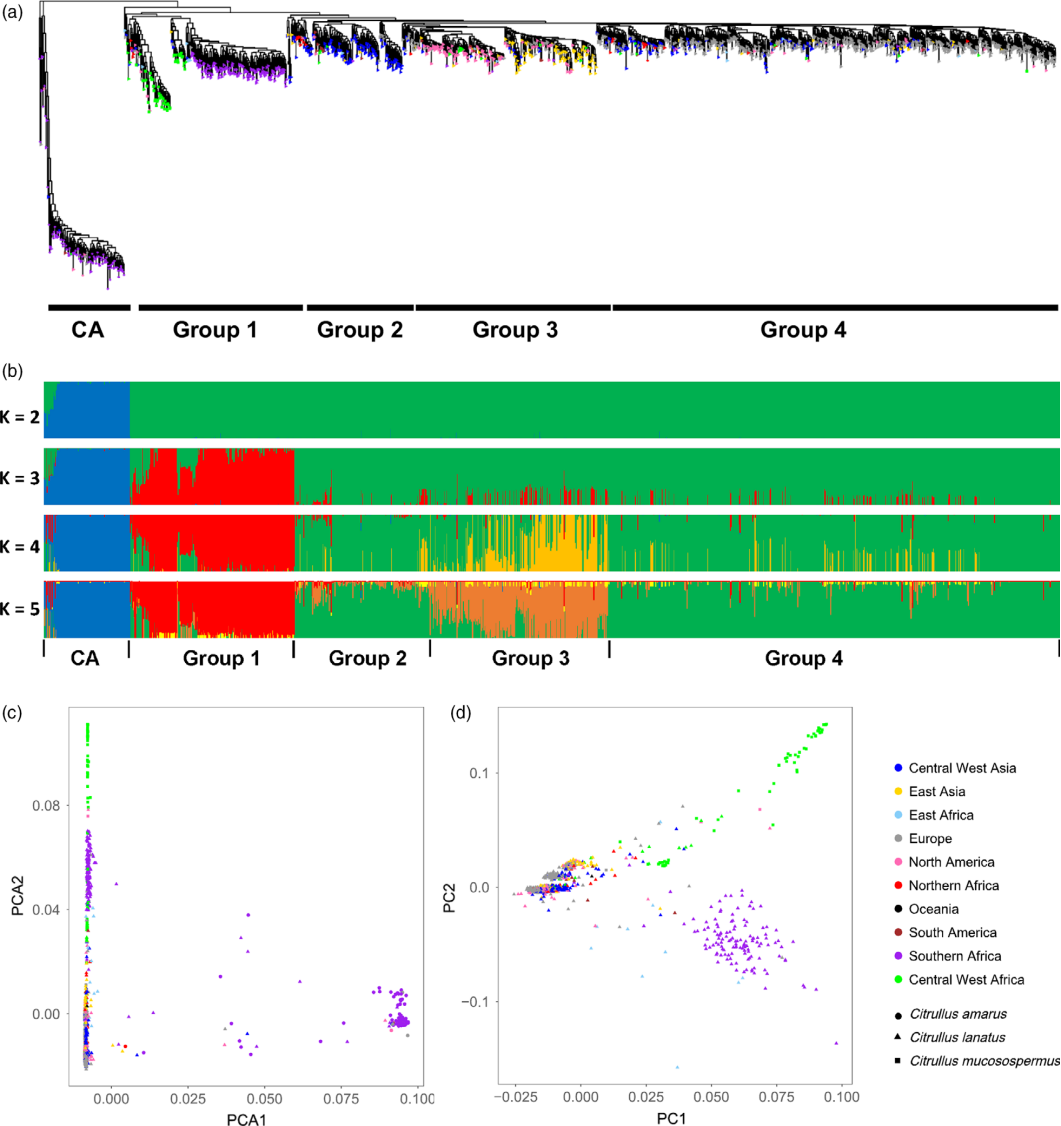
Phylogenetic relationship and population structure of *Citrullus* spp. accessions. (a) Maximum‐likelihood tree of 1367 *Citrullus* spp. accessions. (b) Model‐based clustering analysis with *K* from 2 to 5. Each accession is represented by a vertical bar. Each colour represents one ancestral population, and the length of each coloured segment in each vertical bar represents the proportion contributed by ancestral populations. (c) Principal component analysis of 1367 watermelon accessions with PC1 and PC2 explaining 63.7% and 2.1% of variance, respectively. (d) Principal component analysis of *C. lanatus* and *C. mucosospermus* accessions with PC1 and PC2 explaining 4.6% and 2.3% of variance, respectively.

To infer phylogenetic relationships among the 1367 *Citrullus* accessions (including ‘Charleston Gray’ and ‘97103’ in addition to the 1365 accessions genotyped with GBS), we constructed a maximum‐likelihood tree, which revealed that *C. lanatus* and *C. mucosospermus* accessions exhibited close genetic relationships and were distant from *C. amarus* (Figure 3a). Within *C. lanatus* and *C. mucosospermus*, four major clades were identified (Figure 3a and Table S9). Group 1 included *C. mucosospermus* along with a subset of *C. lanatus* accessions collected in southern Africa, primarily Zimbabwe and Zambia. The remaining groups were all comprised of *C. lanatus* accessions primarily collected in Central and West Asia (Group 2), North America and East Asia (Group 3) and Europe (Group 4). For *C. lanatus* collected in northern Africa, four Sudanese accessions collected in Khartoum (PI 254622, PI 254623 and PI 260733; white flesh and beige seeds) and Darfur (PI 481871; white flesh and black/beige seeds) were closely related to *C. mucosospermus* in Group 1 (Table S9). Eight other accessions collected in northern Africa were in Group 2 (Central and West Asia) in one of the deepest branches of *C. lanatus* outside Group 1, including six collected in Egypt (PI 525090, PI 525086, PI 525087, PI 525084, PI 525091 and PI 525088), one in Sudan (PI 270545, red flesh and black seeds) and one in Algeria (PI 542617; white flesh and black seeds) (Table S9). For accessions collected in East Africa, five out of seven accessions collected in Ethiopia were closely related to accessions collected in Europe (Group 4), and five out of six accessions collected in Somalia were in Group 1 (Table S9). Similarly, accessions collected in Central and West Africa were placed in all four groups (Table S9), with more accessions (mostly collected in Central Africa) clustered with cultivars from North America and East Asia (Group 3). Despite the geographic distance, the North American *C. lanatus* accessions (including ‘Charleston Gray’) and the East Asian cultivars (including ‘97103’) were closely related, although they formed two distinguishable clades in Group 3, reflecting the two major cultivated ecotypes.

Population structure of the 1367 watermelon accessions was investigated. Δ*K* analysis showed that *K *=* *2 ancestral types best explained the structure of this population and separated *C. amarus* from *C. lanatus*/*C. mucosospermus* (Figure S7). At *K *=* *3 or 4, three primary clusters could be observed representing *C. amarus*, the *C. mucosospermus*/southern African *C. lanatus* group and the remaining *C. lanatus* accessions (Figure 3b). At *K *=* *5, a new subgroup emerged, which included North American and East Asian cultivars, while most *C. lanatus* from Central and West Asia and Europe had similar backgrounds (Figure 3b). Principal component analysis (PCA) using all the accessions also revealed a clear separation of *C. amarus* from *C. lanatus* and *C. mucosospermus* (Figure 3c). According to the phylogenetic relationship and genetic background, a few accessions were likely misclassified or had ambiguous identity, including 26 accessions classified as *C. lanatus*, among which 11 might be *C. amarus* and 15 had mixed genetic background, and 14 accessions labelled as *C. amarus* but did not have a typical *C. amarus* ancestry pattern (Table S9). These accessions were excluded from the following genetic diversity and population divergence analyses. PCA using the remaining *C. lanatus* and *C. mucosospermus* accessions illustrated a pattern further separating *C. mucosospermus*,* C. lanatus* collected in southern Africa and the rest of the *C. lanatus* accessions, while the small portion of the total variance explained by the first two principal components further supported the close genetic relationship between *C. lanatus* and *C. mucosospermus* (Figure 3d). Together, the phylogeny, population structure and PCA results all indicated a distant relationship between *C. amarus* and *C. lanatus*/*C. mucosospermus*, and a similar genetic background in *C. mucosospermus* and a group of *C. lanatus* mostly from southern Africa.

### Genetic diversity and population divergence in watermelons

The genetic diversity within *C. amarus* estimated by the average value of genome‐wide nucleotide diversity (π) was 3.375 × 10^−4^, higher than that within *C. mucosospermus* (π = 2.561 × 10^−4^) and those within different *C. lanatus* groups based on geographic locations, ranging from 2.405 × 10^−4^ (Europe) to 3.035 × 10^−4^ (Southern Africa) (Figure S8). Population divergence among different *C. lanatus* groups and *C. mucosospermus* was evaluated by pairwise fixation index (*F*_ST_). Multidimensional scaling visualization of pairwise *F*_ST_ values clearly showed the distinction between *C. mucosospermus* and *C. lanatus*, and between *C. lanatus* collected in southern Africa and the rest of *C. lanatus* groups, and much less divergence among the *C. lanatus* groups outside southern Africa (Figure S8). The weighted pairwise *F*_ST_ values between *C. mucosospermus* and different *C. lanatus* groups, and between the southern Africa *C. lanatus* group and the other groups ranged from 0.286 to 0.428, and from 0.238 to 0.385, respectively, while the values among the other *C. lanatus* groups were much lower, ranging from 0.020 to 0.149 (Table S10). The *F*_ST_ values were largely consistent with relationships of different groups of *Citrullus* accessions inferred from phylogenetic and population structure analyses.

### GWAS for fruit quality and disease resistance traits

**Figure 4:**
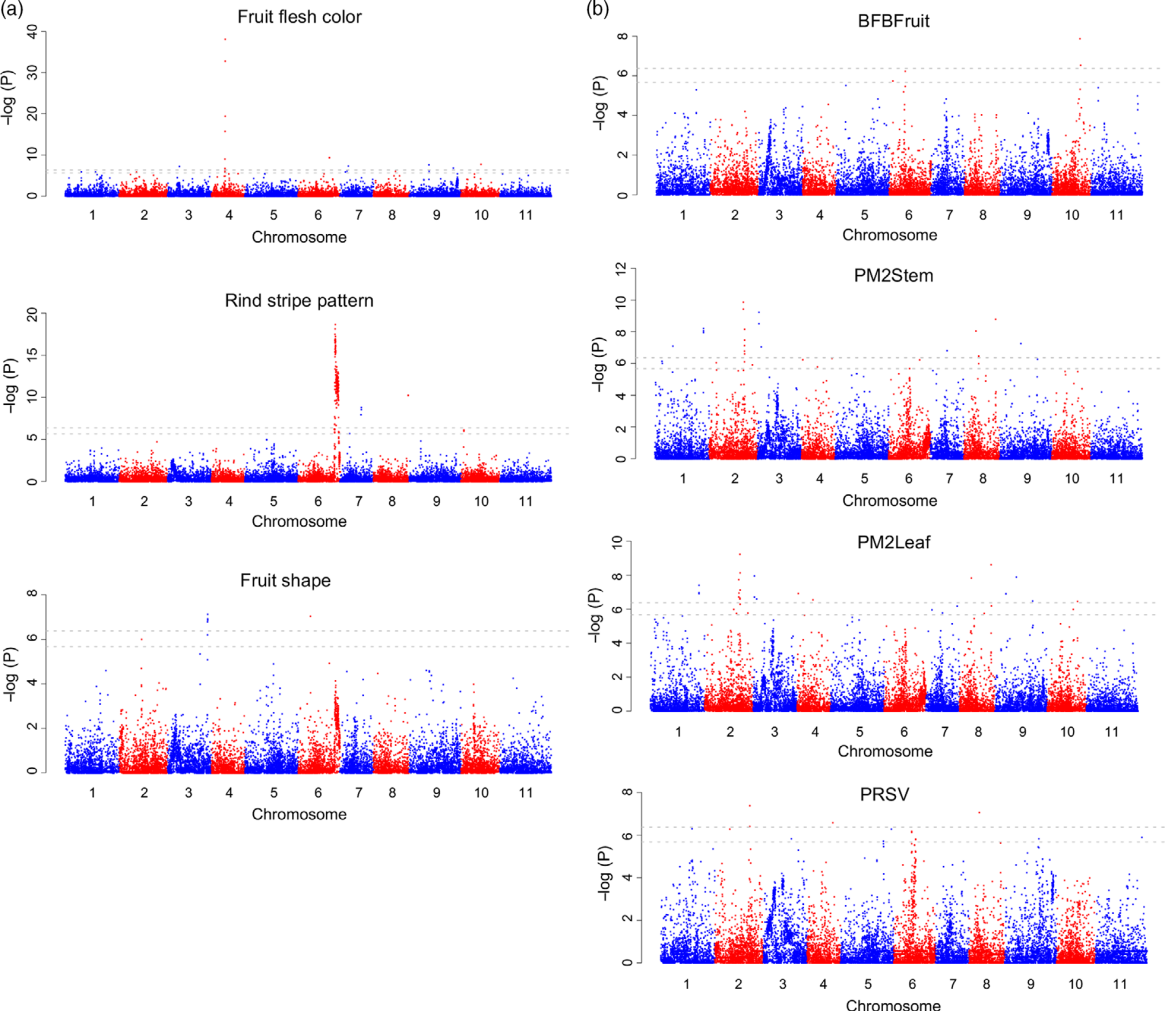
Genome‐wide association studies of fruit quality (a) and disease resistance (b) traits. BFBFruit, resistance to bacterial fruit blotch in fruits; PM2Stem, resistance to powdery mildew race 2W in stem; PM2Leaf: resistance to powdery mildew race 2W in leaf; PRSV: resistance to *Papaya ringspot virus‐watermelon strain*. Gray horizontal dashed lines on the Manhattan plots indicate the Bonferroni significance thresholds of GWAS (−log10(P) of 5.68 and 6.38, corresponding to α = 0.05 and α = 0.01, respectively).

We performed GWAS for several fruit quality and disease resistance traits to identify QTLs underlying these important traits. We collected historical phenotypic data from the NPGS for fruit flesh colour, fruit shape and rind stripe pattern for 788, 864 and 695 accessions, respectively, that were genotyped in this study. We also evaluated the resistance to bacterial fruit blotch (BFB), powdery mildew race 2W and *Papaya ringspot virus‐watermelon strain* (PRSV‐W) on 1125, 1147 and 908 PIs, respectively (Table S11). A group of SNPs on chromosome 4 (at ~15.3–15.8 Mb) were associated with red coloration of fruit flesh (Figure 4a and Table S12), and this genome region contained the *LCYB* gene (chromosome 4: 15 694 446–15 696 571) that converts lycopene to downstream carotenoids (Bang *et al*., 2010). In addition, 14 additional SNPs on other chromosomes were also associated with red flesh colour (Table S12). A peak associated with rind stripe pattern was identified on chromosome 6 (at ~30.2 Mb) (Figure 4a), overlapping with the ‘*S*’ locus that controls the foreground stripe pattern (Park *et al*., 2016). Nine additional SNPs were also significantly associated with rind stripe pattern. Five SNPs on chromosome 3 (at ~31.1 Mb) were associated with elongated fruit shape (Figure 4a and Table S12), which were located near the *ClFS1* gene (~22.6 Kb distance; chromosome 3: 31 086 958–31 090 579) known to control watermelon fruit elongation (Dou *et al*., 2018). Two other SNPs associated with fruit shape were on chromosomes 2 and 6. Two peaks on chromosomes 10 (~32.6–32.9 Mb) and 6 (~10.9 Mb), respectively, were associated with resistance to BFB in fruit (Figure 4b and Table S12). A group of SNPs on chromosome 2 (~29.2–31.0 Mb) were associated with powdery mildew race 2W resistance in stems, and an additional 21 SNPs on other chromosomes were also associated (Figure 4b and Table S12). The same peak on chromosome 2 was also associated with powdery mildew race 2W resistance in leaves, and an overall similar association pattern was observed. For PRSV‐W resistance, 15 significantly associated SNPs were identified on multiple chromosomes (Figure 4b and Table S12).

## Discussion

Watermelon is among the first cucurbit crops to have its genome sequenced, assembled and annotated (Zheng *et al*., 2019). Genome sequence of the East Asian watermelon cultivar, ‘97103’, was released in 2013 (Guo *et al*., 2013). To complement the ‘97103’ reference genome, and to capture genome information specifically present in the American watermelon ecotype, we assembled and annotated the genome of the principal American cultivar ‘Charleston Gray’, performed detailed characterization of sequence variations between ‘Charleston Gray’ and ‘97103’, and identified those overlapping with known watermelon fruit trait QTLs, which may underlie the phenotypic differences between the two cultivated ecotypes. Comparative analyses suggested that the assembled ‘Charleston Gray’ genome had higher continuity and quality than that of ‘97103’. The high‐quality ‘Charleston Gray’ assembly will facilitate its use as a reference for integrative genetic‐genomic studies aiming to identify QTLs associated with important agronomical traits.

We characterized the genetic composition of 1365 watermelon PIs maintained in the NPGS, belonging to three *Citrullus* species, *C. lanatus*,* C. mucosospermus* and *C. amarus*. The latter two are of particular interest because of the presence of potential genes/alleles associated with fruit quality or resistance to major cultivated watermelon diseases (Levi *et al*., 2013). In agreement with the known narrow genetic background in dessert watermelon, all *C. lanatus* groups from different geographic regions displayed low genetic diversity, with that of the southern Africa group being slightly higher, consistent with southern Africa being the likely centre of origin of *Citrullus* (Chomicki and Renner, 2015; Paris, 2015).

Our sampling included a large number of accessions collected in major dessert watermelon production areas and geographic regions where wild and primitive watermelons exist. This enabled us to reveal phylogenetic relationships among watermelon accessions collected in different geographic regions that may shed light on the domestication and dispersal of dessert watermelons. The distant relationship between *C. amarus* and *C. lanatus*/*C. mucosospermus* and their distinct genetic backgrounds revealed in the current study are consistent with previous genetic characterization of a few resequenced accessions (Guo *et al*., 2013). This result indicates that the dessert watermelon is unlikely to be descended from *C. amarus* in southern Africa, which is further supported by the evidence of genome organization differences based on rDNA chromosome landmarks (Guo *et al*., 2013; Reddy *et al*., 2013) and the finding that the emergence of dessert watermelon in Egypt predated the time when farming began in southern Africa (Paris, 2015). It has been hypothesized that the dessert watermelon was domesticated in northeastern Africa and a wild population of ‘*cordophanus*’ watermelons in Sudan with non‐bitter fruits and white pulp may be the living representatives of the progenitor of the cultivated dessert watermelon (Paris, 2015; Renner *et al*., 2017). Four Sudanese *C. lanatus* accessions bearing such fruit phenotypes, including two (PI 481871 and PI 254622) considered as dessert watermelon (Paris, 2015), are closely related to *C. mucosospermus* collected from Liberia, Ghana, Nigeria and Zaire, suggesting that *C. lanatus* accessions from Sudan and *C. mucosospermus* from central/West Africa could be derived from the same ancestral population; one selected for seed traits, and the other domesticated for fruit flesh characteristics. The placement of eight northern African *C. lanatus* accessions (six from Egypt) in one of the deepest branches of cultivated dessert watermelon groups on the phylogenetic tree (Figure 3a) further supports the hypothesis that modern dessert watermelons originated in northeastern Africa. Accessions from Central/West Asia are also located in relatively deep branches on the phylogenetic tree, suggesting that dessert watermelons might have been introduced from Africa to Central/West Asia, and then dispersed to the rest of the world. The accessions from East Asia and North America are closely related and yet clearly distinguishable, reflecting the differences in recent breeding efforts in the two areas. East Asian and North American accessions share a unique genetic background different from that of most current accessions in Central/West Asia and Europe, and a small group of European accessions (mostly from Hungary) were found in the deepest branch in the East Asia/North America clade, suggesting that watermelons from East Asia and North America might be derived from a single ancestral group and could be distributed through Europe. By including a large collection of *C. lanatus* accessions collected in southern Africa, our analyses showed a close relationship between *C. lanatus* collected in southern Africa and *C. mucosospermus* in Mali and Senegal. The *C. lanatus* accessions collected in southern Africa display various flesh colours and sweetness levels (https://npgsweb.ars-grin.gov/gringlobal/descriptors.aspx) and have a unique genetic background different from the other *C. lanatus* accessions collected in regions other than southern Africa, suggesting parallel evolution for increased flesh sugar content and coloration in different *C. lanatus* populations, or introduction of alleles controlling these trait from more improved cultivars to the southern African accessions. Knowledge of causal genes underlying domestication traits and distribution of functionally diverse alleles in different watermelon populations is needed to better demonstrate the evolutionary route of dessert watermelons.

The enhancement of disease resistance has been a major focus of current watermelon improvement programmes. Using the GBS SNP makers combined with GWAS, potential QTLs for resistance to BFB, powdery mildew and PRSV‐W were identified. BFB, caused by *Acidovorax citrulli*, is a devastating disease of most cucurbit crops including watermelon. Numerous BFB outbreaks have occurred in watermelon growing regions in the United States and in countries throughout the world. Recently, using a recombinant inbred line population, Branham *et al*. (2019) identified six QTLs significantly associated with foliar BFB resistance. Three of the QTLs (*qAc‐1.1*,* qAc‐2.1* and *qAc‐8.1*, on chromosomes 1, 2 and 8, respectively) were consistent in multiple tests and explained the highest proportion of variation. Given the low heritability and large environmental component of disease response to *A. citrulli*, the three QTLs (*qAc‐1.1*,* qAc‐2.1* and *qAc‐8.1*) identified in *C. amarus* and the two additional QTLs identified in this study on chromosomes 10 (~32.6–32.9 Mb) and 6 (~10.9 Mb) in *C. lanatus*/*C. mucosospermus* can be further explored for marker development and used to pyramid genes/loci for enhanced BFB resistance in elite watermelon cultivars.

Powdery mildew is a major disease of cucurbit crops, caused by the fungus *Podosphaera xanthii*. In the United States, two distinct powdery mildew races, 1W and 2W, are known for watermelon, and several cultivars and wild species highly resistant to these races have been identified (Davis *et al*., 2007; Tetteh *et al*., 2010). Recently, Kim *et al*. (2015) identified a major QTL (*pmr2*.*1*) for resistance to powdery mildew race 1W on chromosome 2 that explained 80.0% of the phenotypic variation. In the present study, GWAS analysis identified a major peak associated with powdery mildew race 2W resistance also on chromosome 2 (~29.2–31.0 Mb) overlapping with QTL *pmr2*.*1,* and additional SNPs on chromosomes 3, 6, 8, 9 and 10 associated with race 2W resistance (Figure 4b and Table S12). These results indicate the possibility that the QTL on chromosome 2 might be associated with resistance to both powdery mildew races 1W and 2W.

PRSV‐W is an important potyvirus causing serious economic damage to the watermelon crop. Resistance to PRSV‐W has been identified, and genetic inheritance mode of this important potyvirus in three *C. amarus* accessions (PI 244017, PI 244019 and PI 485583) has been determined, suggesting that the resistance is controlled by a single recessive gene (Guner *et al*., 2018). In contrast, a genetic inheritance study of PRSV‐W resistance in *C. lanatus* PI 595201 indicated that the inheritance of PRSV‐resistance is complex with additive genetic effects, and is controlled by multiple genes (de Azevedo *et al*., 2012). The GWAS analysis here, using the *C. lanatus*/*C. mucosospermus* PIs, identified SNPs on multiple chromosomes with significant associations with PRSV‐W resistance (Figure 4b and Table S12). These results suggest that resistance to PRSV‐W in *C. lanatus* might be controlled by several genes with additive effects while in *C. amarus* the resistance is controlled by a single gene.

GWAS analyses in the present study also successfully identified genomic regions known to underlie fruit quality traits such as flesh colour, fruit shape and rind pattern, and additional novel SNPs highly associated with these traits. The QTLs identified in this study together with QTLs identified in recent genetic inheritance studies provide a useful platform for the development of molecular markers for use in breeding programmes aiming to enhance disease resistance and fruit quality in elite watermelon cultivars.

## Experimental procedures

### Plant materials

For genome and transcriptome sequencing of ‘Charleston Gray’, seeds were obtained from a line descended from the original ‘Charleston Gray’ through self‐pollination over several generations at the U.S. Vegetable Laboratory (USVL) and germinated in a greenhouse at the USVL. For GBS, a total of 1365 PIs, including 1211 *C. lanatus*, 52 *C. mucosospermus* and 102 *C. amarus,* were obtained from self‐pollinated plants (one generation) and grown in the greenhouse at North Carolina State University.

### Construction and sequencing of genomic and RNA‐Seq libraries

Genomic DNA was extracted from young fresh leaves of ‘Charleston Gray’ using the QIAGEN DNeasy Plant Mini Kit (QIAGEN, Valencia, CA) following the manufacturer's instructions. Two paired‐end genomic libraries with insert sizes of 400 bp and 1 kb, respectively, were prepared using the Genomic DNA Sample Prep kit (Illumina, San Diego, CA) according to the manufacturer's protocol, and sequenced on an Illumina GAIIx platform. The 400‐bp library was sequenced additionally on a HiSeq 2500 system. Another library with insert size of 400 bp was constructed with the DNA Library Preparation kit (Kapa Biosystems, Wilmington, MA) without PCR amplification, and sequenced on an Illumina MiSeq system. Four mate‐pair libraries with insert sizes of 3–5, 8–10 (2 libraries) and 15–20 kb were prepared with the Nextera Mate Pair Sample Preparation kit (Illumina), and sequenced on a HiSeq 2500 system. All libraries were sequenced with the paired‐end mode.

Total RNA was extracted from fruit flesh tissues of ‘Charleston Gray’ at 11, 20, 30 and 40 days after pollination, with two biological replicates for each stage, using the QIAGEN RNeasy Plant Mini Kit (QIAGEN). RNA‐Seq libraries were constructed using the NEB Next Ultra^TM^ RNA Library Prep Kit (NEB, Beverly, MA) and sequenced on the Illumina HiSeq 2500 system with the paired‐end mode.

### *De novo* genome assembly

Duplicated read pairs, defined as having identical bases at positions of 14–90 in both left and right reads, were collapsed into unique read pairs. The non‐redundant reads were processed with Trimmomatic (Bolger *et al*., 2014) to remove adaptors and low‐quality sequences. The mate‐pair reads were processed with the ShortRead package (Morgan *et al*., 2009) to remove junction adaptors. The resulting reads were assembled into scaffolds using SOAPdenovo2 (Luo *et al*., 2012). Gaps in the scaffolds were filled with GapCloser (Luo *et al*., 2012). Pilon (Walker *et al*., 2014) was used to correct base errors, fix mis‐assemblies and fill additional gaps. The assembly was then aligned to NCBI non‐redundant nucleotide database using BLASTN with an E‐value cut‐off of 1e‐5. Scaffolds with more than 95% of their length similar to sequences of microorganisms, mitochondria or chloroplasts were considered contaminants and removed. Redundant scaffolds with sequence identity higher than 99% and more than 95% of their length covered by other scaffolds were removed.

### Transposable element annotation and gene prediction

Long terminal repeat retrotransposon (LTR‐RT) and miniature inverted‐repeat transposable element (MITE) libraries were *de novo* constructed by screening the ‘Charleston Gray’ genome using LTRharvest (Ellinghaus *et al*., 2008) and MITE‐Hunter (Han and Wessler, 2010), respectively. After masking the assembly with these libraries using RepeatMasker (http://www.repeatmasker.org), we further searched for repeat elements in the unmasked sequences using RepeatModeler (http://www.repeatmasker.org/RepeatModeler.html). All the identified repetitive sequences were combined into a single repeat library and compared against the Swiss‐Prot database (Magrane and UniProt Consortium, 2011). Sequences that matched non‐TE proteins in the database were removed. TEs were classified using REPCLASS (Feschotte *et al*., 2009). The classified repeat library was then used to identify TEs in the ‘Charleston Gray’ genome with RepeatMasker.

The repeat‐masked ‘Charleston Gray’ assembly was used for gene prediction with MAKER (Cantarel *et al*., 2008) by integrating evidences from *ab initio* gene prediction, transcript mapping and protein homology to define confident gene models. SNAP (Korf, 2004) and AUGUSTUS (Stanke *et al*., 2006) were used for *ab initio* gene predictions. Two transcriptome assemblies from fruit RNA‐Seq data were obtained using Trinity (Grabherr *et al*., 2011) with the *de novo* mode and the genome‐guided mode, respectively, and aligned to the ‘Charleston Gray’ assembly using PASA2 (Haas, 2003). The resulting alignments were used as the transcript evidence. Protein sequences from Arabidopsis, watermelon, cucumber and melon, as well as the UniProt (Swiss‐Prot plant division) database, were aligned to the ‘Charleston Gray’ genome using Spaln (Iwata and Gotoh, 2012) to provide protein homology evidence. For gene annotation, protein sequences of the predicted ‘Charleston Gray’ genes were compared against the Arabidopsis protein and UniProt (Swiss‐Prot/TrEMBL) databases using BLAST, as well as the InterPro database using InterProScan (Jones *et al*., 2014). Blast2GO (Conesa *et al*., 2005) was used to obtain gene ontology (GO) annotations.

### Synteny analysis

To identify syntenic regions between the ‘Charleston Gray’ and ‘97103’ genomes, protein sequences from the two cultivars were aligned using BLASTP, and high‐confidence collinear blocks were determined using MCScanX with an E‐value cut‐off of 1e‐10 (Wang *et al*., 2012). Ks values of orthologous gene pairs were calculated using the Yang–Nielsen algorithm implemented in PAML (Yang, 1997). LAST (v869; Kielbasa *et al*., 2011) was used to identify unique best alignments between the genomes of ‘Charleston Gray’ and ‘97103’.

### Identification of SNPs and SVs

To detect SNPs and small indels between ‘97103’ and ‘Charleston Gray’, paired‐end genomic reads of ‘97103’ (Guo *et al*., 2013) were mapped to the ‘Charleston Gray’ genome using BWA (version 0.6.2) (Li and Durbin, 2009). Uniquely mapped reads were kept for variant detection. Genotypes were assigned to each genomic position based on the alignment mpileup files generated by SAMtools (Li *et al*., 2009). SNPs and small indels were then identified if they were supported by at least four mapped reads. To detect large SVs (≥50 bp), genome sequences of ‘97103’ and ‘Charleston Gray’ were aligned using Minimap2 (Li, 2018), and based on the alignment indels and tandem/repeat expansions/contractions were identified using Assemblytics (Nattestad and Schatz, 2016). To confirm the identified SVs, reads generated from one genome were aligned to the opposite genome using bowtie (Langmead *et al*., 2009) allowing one mismatch. SVs with junction sites at both ends having no more than five spanning reads were considered highly confident. Meantime, indels (≥50 bp) were also detected with SpeedSeq (Chiang *et al*., 2015) based on ‘97103’ genomic reads mapped to the ‘Charleston Gray’ genome and ‘Charleston Gray’ reads mapped to the ‘97103’ genome. Sequences flanking the identified indels (five kb on each side) from one genome were aligned to the other genome using BLAST with E‐value <1e‐10 and sequence identity >90%. Indels with flanking sequences aligned to the proper positions on the other genome were considered highly confident. The two high‐confidence sets of SVs by reference sequence comparison and read mapping, respectively, were integrated, and those spanning any gap regions in the assembled genomes were removed.

### DNA extraction for GBS

About 100 mg fresh leaf tissue was collected from a young seedling representing each PI. The leaf tissue was freeze‐dried and then ground to a fine powder using 5/32” stainless steel balls (AbbottBall, West Hartford, CT) in a Retsch Mixer Mill (Retsch, Newtown, PA). DNA was isolated using the Plant DNA DS Kit (Omega Bio‐Tek, Norcross, GA). The DNA was quantified with the Quant‐iT PicoGreen dsDNA Kit (Invitrogen, Carlsbad, CA), and its quality was checked by electrophoresis of undigested and *Hind*III‐digested DNA on agarose gels.

### GBS analysis and SNP calling

DNA samples of all PI accessions were subjected to GBS analysis following the protocol described in Elshire *et al*. (2011) using the *ApeK*I restriction enzyme (NEB). GBS libraries were sequenced on a HiSeq 2500 system to obtain reads with lengths of 101 bp. The TASSEL 5.0 GBS Discovery Pipeline (Glaubitz *et al*., 2014) was used for SNP identification using the ‘Charleston Gray’ genome as the reference. Tags were identified from raw reads possessing a barcode and a restriction enzyme cut site using GBSSeqToTagDBPlugin with parameters ‘‐kmerLength 90 ‐minKemrL 30 ‐mnQS 10 ‐c 10 ‐maKmerNum 200000000’. Tags supported by at least ten reads were retrieved and reformatted using TagExportToFastqPlugin, and mapped to the ‘Charleston Gray’ genome using BWA (v0.7.13; Li *et al*., 2009) with default parameters. Based on the alignments, positions of aligned tags were determined using SAMtoGBSdbPlugin, and SNPs were identified from the aligned tags using DiscoverySNPCallerPluginV2 with default parameters. SNPs were filtered using VCFtools (Danecek *et al*., 2011) to keep those that were biallelic and had missing rate ≤ 50% and MAF ≥1%.

### Phylogenetic and population genomic analyses

Biallelic SNPs with MAF ≥1% and missing rate ≤50% were further filtered to remove those in high linkage disequilibrium (LD) blocks using SNPhylo (v20140701; Lee *et al*., 2014) with the parameter ‘‐l 0.1’, and the remaining SNPs were used in phylogenetic and population structure analyses. A maximum‐likelihood tree was constructed using IQTREE with 1000 bootstraps (v1.6.8; Nguyen *et al*., 2015), using *C. amarus* accessions as the outgroup. ggtree (v1.10.5; Yu *et al*., 2017) was used to visualize the phylogenetic tree.

PCA was performed using Plink (v1.9; Purcell *et al*., 2007). Population structure analysis was performed using STRUCTURE (v2.3.4; Falush *et al*., 2003). To determine the most likely cluster number, STRUCTURE analyses were run 20 times for each *K* value ranging from 2 to 20, using 3715 SNPs filtered by LD information using SNPhylo described above, with an admixture model. After the best *K* (*K *=* *2) was determined, population structure of the watermelon accessions was inferred using fastStructure (v1.0; Raj *et al*., 2014) with all SNPs for each *K* (*K *=* *2‐5).

Nucleotide diversity (π) and population fixation index (*F*_ST_) were calculated using vcftools (v 0.1.15; Danecek *et al*., 2011) using unfiltered raw SNPs. The average nucleotide diversity was calculated as the sum of nucleotide diversity at each site divided by the total bases covered by GBS reads in the genome.

### Phenotypic data collection and disease symptom rating

Historical phenotypic data for watermelon fruit quality traits were downloaded from the GRIN database (https://npgsweb.ars-grin.gov/gringlobal/descriptors.aspx). Fruit flesh colour was rated as red (red and pink) and non‐red (green, white, yellow and orange). Fruit shape was rated as round and elongated (oblate, oblong and elongated). Rind stripe pattern was rated as striped and solid. For PRSV‐W disease screening, young watermelon seedlings were rated at 14, 21 and 28 days post‐inoculation (DPI), and the average of the three ratings was used. BFB disease data were collected from young fruits at 21 DPI. Powdery mildew race 2W disease data were collected from young seedlings at 14 and 28 DPI, and the average of the two ratings was used. All three diseases were rated using a visual scale of 0‐9, where 0 = no symptoms and 9 = dead plant. Phenotypic data for accessions genotyped in the present study were used for GWAS.

### Genome‐wide association studies

*Citrullus lanatus* and *C. mucosospermus* accessions were used in GWAS. A Balding–Nichols kinship matrix (Balding and Nichols, 1995) constructed using the 57 294 unfiltered biallelic SNPs was used to correct population structure. Missing genotypes were imputed using the k‐nearest neighbour algorithm implemented in fillGenotype (Huang *et al*., 2010) with an optimal combination of parameters (w = 30, k = 9, p = −9, r = 0.8) determined based on filling rate and imputation accuracy (Table S13). Each combination of the following parameters: w (20, 30, 50, 65, 80), p (−3, −5, −7, −9), k (3, 5, 7, 9), and r (0.65, 0.7, 0.75, 0.8), was tested with accessions having lowest missing rates in each of the two *Citrullus* species (*C. mucosospermus* PI 306780, and *C. lanatus* PI 635626), and 10%, 20% and 30% SNP sites were randomly masked as missing genotypes for imputing. Only biallelic imputed SNPs within *C. lanatus* and *C. mucosospermus* accessions (a total of 24 065 SNPs) were used for GWAS. GWAS were performed using the linear mixed model implemented in EMMAX (Kang *et al*., 2010). Genome‐wide significance thresholds of GWAS were determined using the Bonferroni correction at α = 0.05 and α = 0.01 for significant and extremely significant associations, respectively, as described in Li *et al*. (2012).

## Supporting information


**Figure S1 **K‐mer distribution of Illumina genomic sequencing reads of ‘Charleston Gray’.
**Figure S2** Syntenic orthologous gene blocks between ‘Charleston Gray’ and ‘97103’.
**Figure S3** Collinearity between the ‘Charleston Gray’ and ‘97103’ genomes.
**Figure S4** Collinearity between ‘Charleston Gray’ and ‘97103’ chromosome 1.
**Figure S5** Size distribution of indels between genomes of ‘Charleston Gray’ and ‘97103’.
**Figure S6** SNP density across the 11 watermelon ‘Charleston Gray’ chromosomes.
**Figure S7** Plot of Δ*K* values with *K* from 2 to 20 in the STRUCTURE analysis for the 1367 watermelon accessions using GBS SNPs.
**Figure S8** Multidimensional scaling of pairwise *F*_ST_ between *C. mucosospermus* (CM) and *C. lanatus* accessions from different geographic regions.

**Table S1** Summary of ‘Charleston Gray’ genome sequencing data.
**Table S2** Statistics of ‘Charleston Gray’ pseudomolecules.
**Table S3** Mapping of RNA‐Seq reads and ESTs to the ‘Charleston Gray’ genome.
**Table S4** Assessment of ‘Charleston Gray’ genome assembly completeness by BUSCO.
**Table S5** Summary of repeat sequences in the ‘Charleston Gray’ genome.
**Table S6** Summary of SNPs and small indels between ‘Charleston Gray’ and ‘97103’.
**Table S7** Structural variations between ‘Charleston Gray’ and ‘97103’.
**Table S8** Structural variations between ‘Charleston Gray’ and ‘97103’ overlapping with known QTLs.
**Table S9** List of the 1365 watermelon accessions from the NPGS collection.
**Table S10** Weighted pairwise *F*_ST_ values between *C. mucosospermus* and *C. lanatus* accessions from different geographic regions.
**Table S11** Phenotypic data for GWAS.
**Table S12** SNPs significantly associated with agronomic traits identified through GWAS.
**Table S13** Evaluation of the SNP imputation using KNN under the optimal parameters ‘w = 50, k = 7, p = ‐9, r = 0.8’.

## Data Availability

The genome sequence of ‘Charleston Gray’ has been deposited at DDBJ/ENA/GenBank under the accessions CP026477‐CP026488. Raw genome and transcriptome sequencing reads have been deposited into the NCBI sequence read archive (SRA) under accessions SRP183199 and SRP183523, respectively. The genome sequence of ‘Charleston Gray’ and the GBS SNPs are also available at the Cucurbit Genomics Database (http://cucurbitgenomics.org; Zheng *et al*., 2019).
