# Supplementary material for: Genome of ‘Charleston Gray’, the principal American watermelon cultivar, and genetic characterization of 1,365 accessions in the U.S. National Plant Germplasm System watermelon collection
Source: Plant Biotechnol J. 2019 May 7;17(12):2246–58. doi: 10.1111/pbi.13136 (PMC6835170; doi:10.1111/pbi.13136)
Supplement: Supplementary file 1 — Figure S1 K‐mer distribution of Illumina genomic sequencing reads of ‘Charleston Gray’. Figure S2 Syntenic orthologous gene blocks between ‘Charleston Gray’ and ‘97103’. Figure S3 Collinearity between the ‘Charleston Gray’ and ‘97103’ genomes. Figure S4 Collinearity between ‘Charleston Gray’ and ‘97103’ chromosome 1. Figure S5 Size distribution of indels between genomes of ‘Charleston Gray’ and ‘97103’. Figure S6 SNP density across the 11 watermelon ‘Charleston Gray’ chromosomes. Figure S7 Plot of ΔK values with K from 2 to 20 in the STRUCTURE analysis for the 1367 watermelon accessions using GBS SNPs. Figure S8 Multidimensional scaling of pairwise F ST between C. mucosospermus (CM) and C. lanatus accessions from different geographic regions. [file PBI-17-2246-s001.pdf]

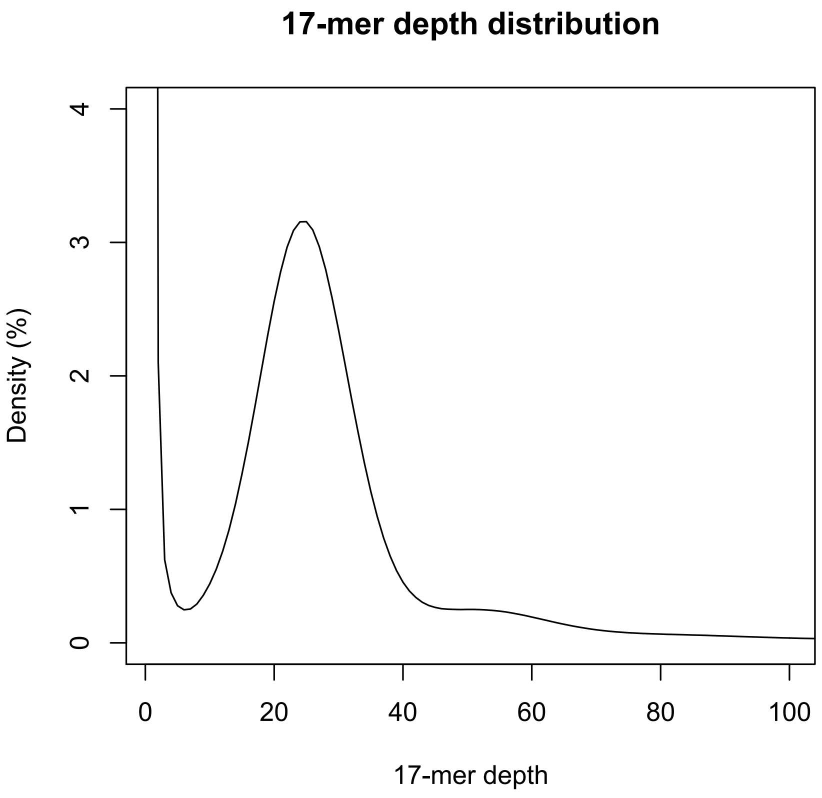


**Figure S1.** K-mer distribution of Illumina genomic sequencing reads of 'Charleston Gray'.

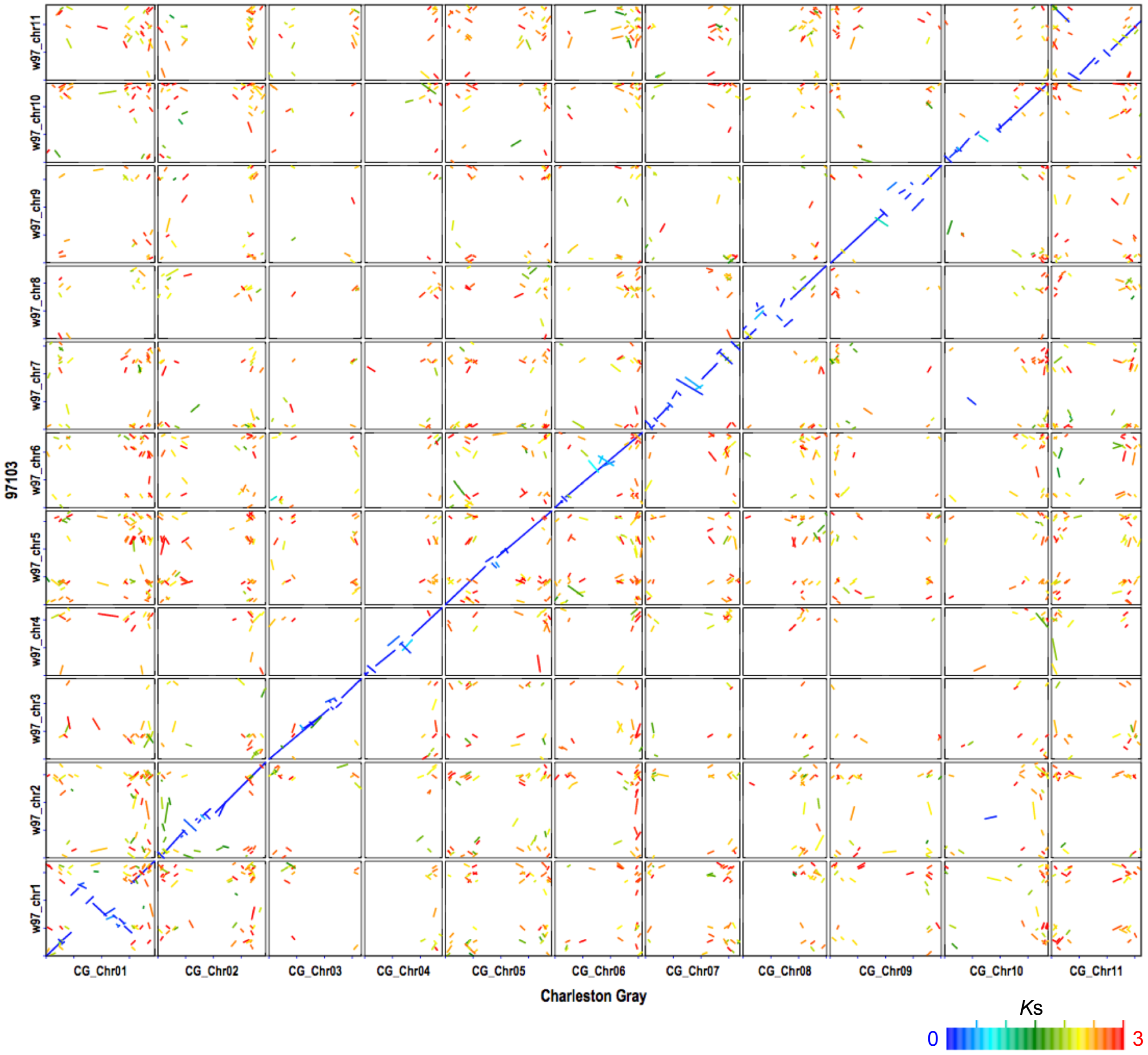


**Figure S2.** Syntenic orthologous gene blocks between 'Charleston Gray' and '97103'. The average *K*s values between orthologous genes in the syntenic blocks are indicated by colors.

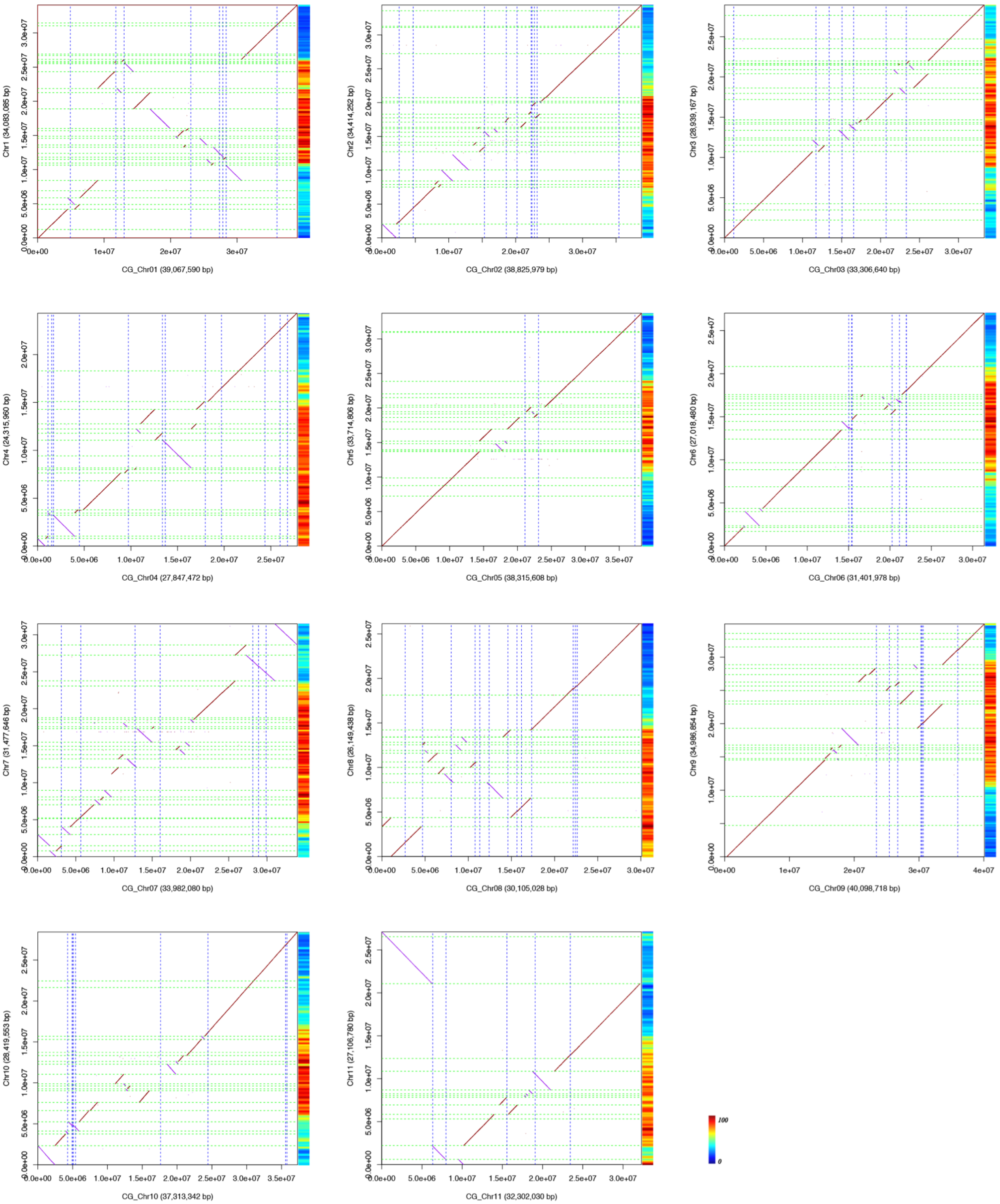


**Figure S3.** Collinearity between the 'Charleston Gray' and '97103' genomes. Boundaries of scaffolds are indicated by green and blue dotted lines for '97103' and 'Charleston Gray', respectively. Heatmaps of transposable element (TE) distribution on 97103 chromosomes are shown on the right (adapted from Guo et al., 2013) to indicate centromeres. The scale bar represents TE density.

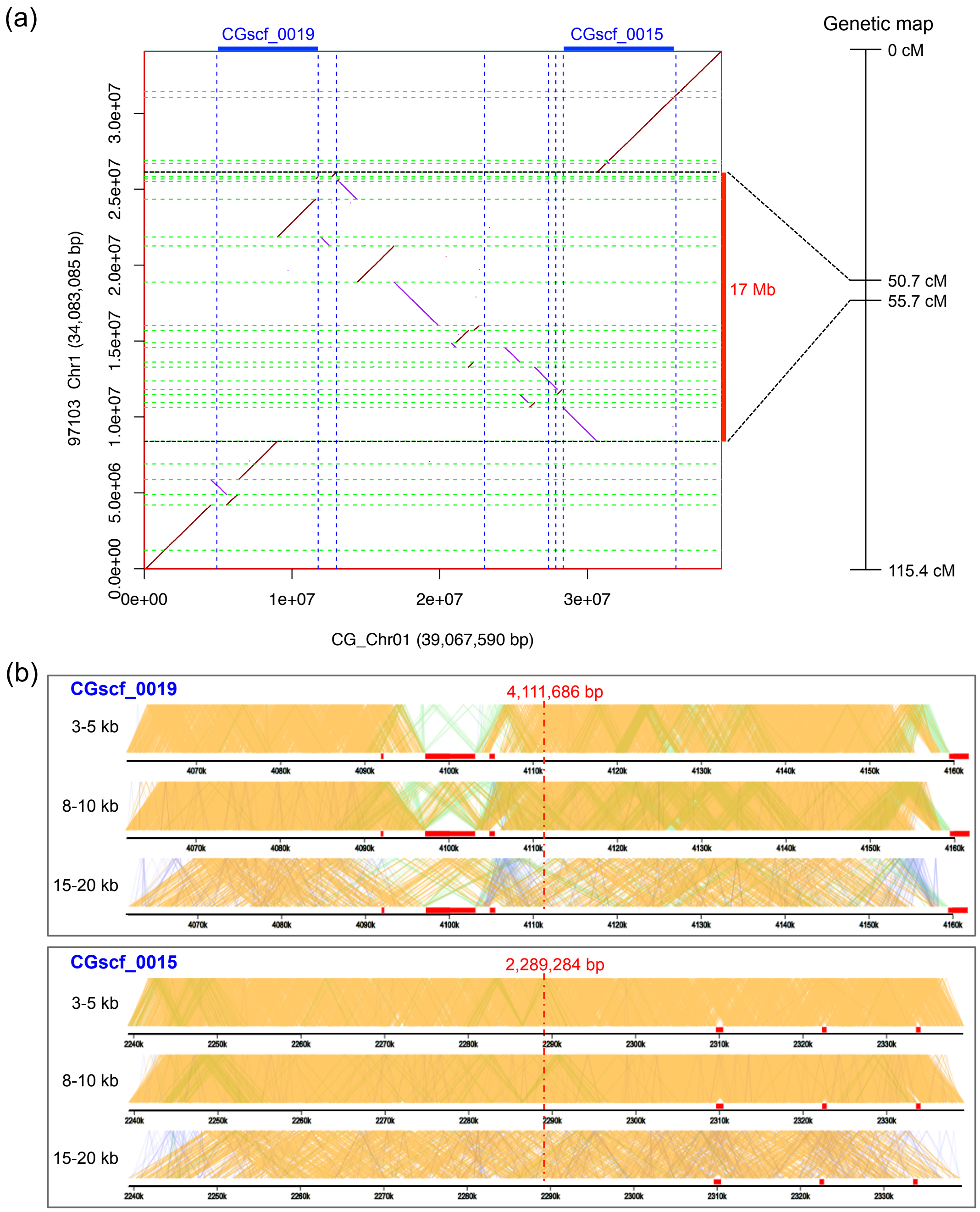


**Figure S4.** Collinearity between 'Charleston Gray' and '97103' chromosome 1. (a) Syntenic dot plot of 'Charleston Gray' and '97103' chromosome 1. Boundaries of scaffolds are indicated by green and blue dotted lines. A diagram of the 97103 genetic map is shown on the right. Two 'Charleston Gray' scaffolds harboring potential "inversion breakpoints", CGscf_0019 and CGscf_0015, are indicated by blue bars. (b) Mate-pair read coverage on CGscf_0019 and CGscf_0015. Mate-pair read mapping results did not support breakage of these scaffolds. The dotted red lines indicated the false inversion breakpoints. Uniquely aligned read pairs with correct insert size and insert size larger or smaller than expected are indicated by orange and red or blue lines, respectively. Reads aligned to multiple locations are shown by green lines. Red bars indicated the gaps in the scaffolds.

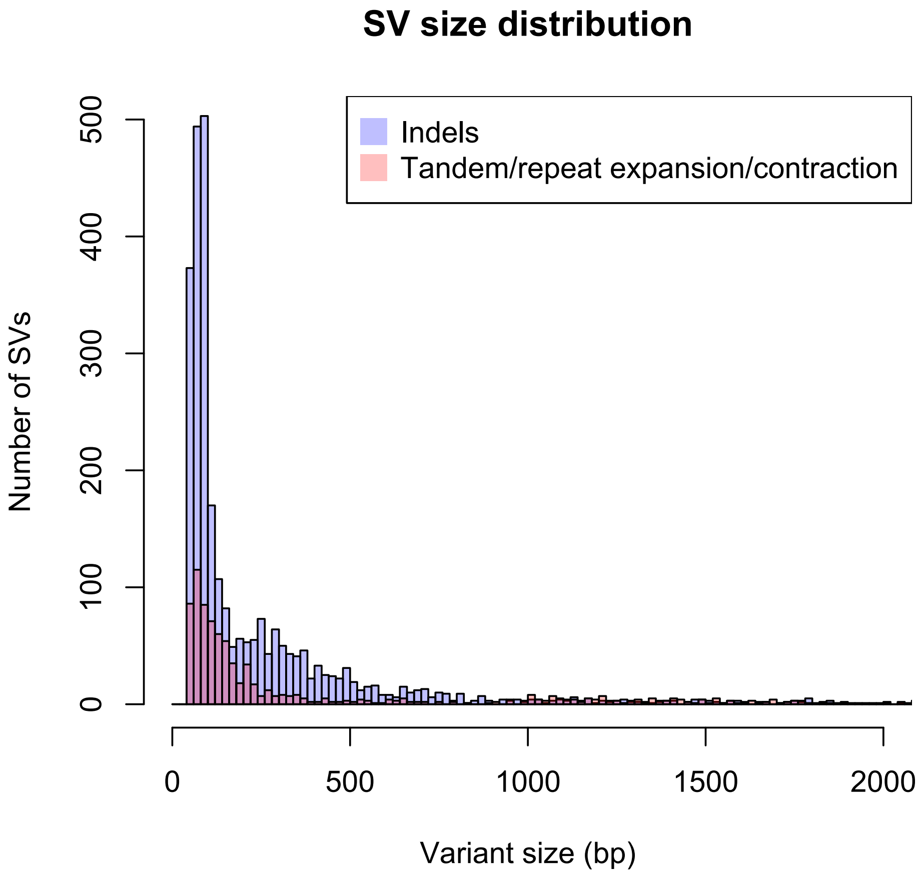


**Figure S5.** Size distribution of SVs between genomes of ‘Charleston Gray’ and ‘97103’.

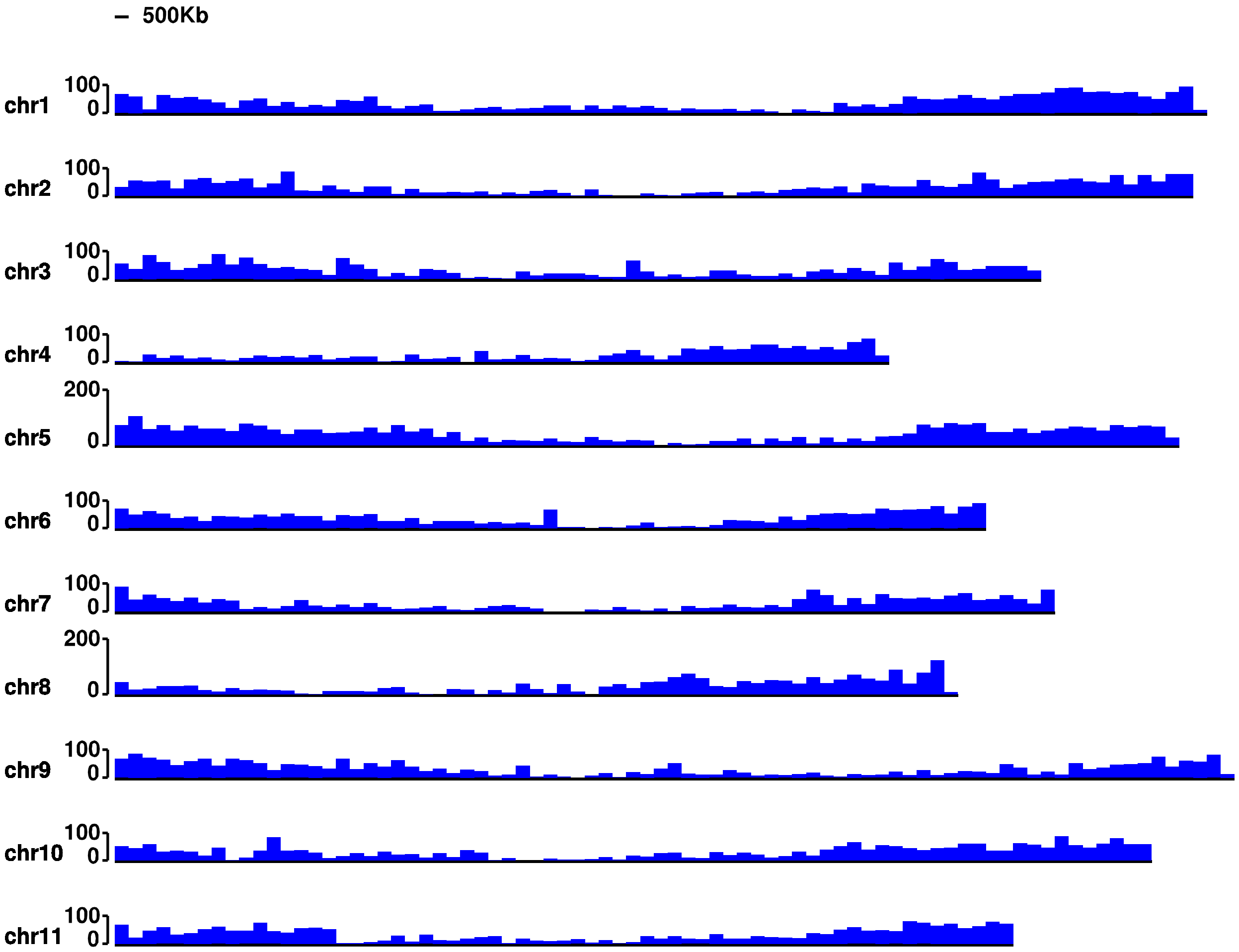


**Figure S6.** SNP density across the 11 watermelon 'Charleston Gray' chromosomes. Number of GBS-SNPs in each 500-Kb non-overlapping window is shown.

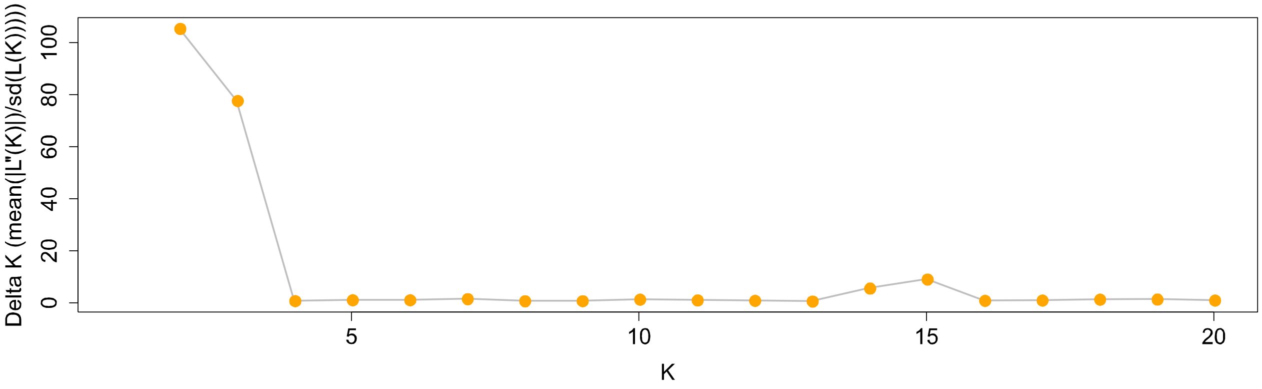


**Figure S7.** Plot of $\Delta K$ values with $K$ from 2 to 20 in the STRUCTURE analysis for the 1,367 watermelon accessions using GBS SNPs.

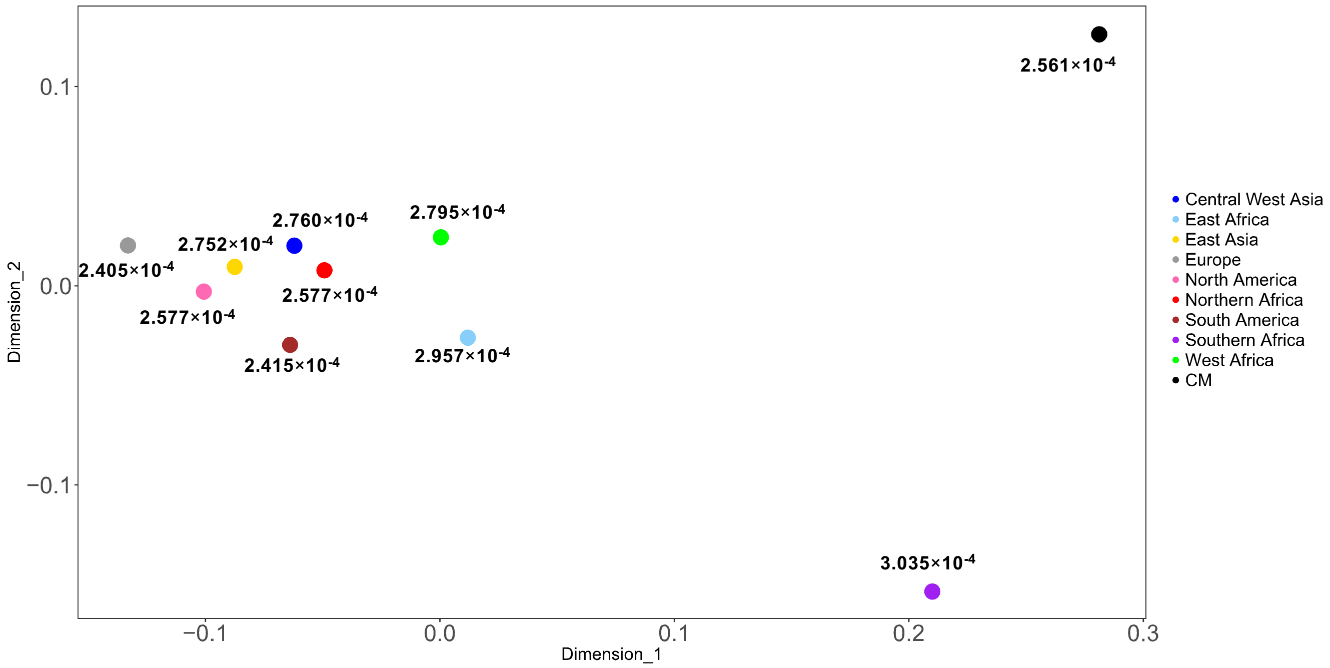


**Figure S8.** Multidimensional scaling of pairwise $F_{ST}$ between *C. mucosospermus* (CM) and *C. lanatus* accessions from different geographic regions. $F_{ST}$ values were transformed into two-dimensional values by multidimensional scaling using the cmdscale function in R. Number near each dot indicates the nucleotide diversity ($\pi$) within the corresponding group.
